# Prevalence of traumatic events and PTSD symptoms among secondary school students in Baghdad

**DOI:** 10.3402/ejpt.v5.23928

**Published:** 2014-11-11

**Authors:** Ashraf Al-Hadethe, Nigel Hunt, Shirley Thomas, Abdulgaffar Al-Qaysi

**Affiliations:** 1 Division of Psychiatry and Applied Psychology, School of Medicine, The University of Nottingham, Nottingham, UK; 2 Division of Rehabilitation and Ageing, School of Medicine, The University of Nottingham, Nottingham, UK; 3 Department of Education & Psychology, College of Education for Women, Baghdad University, Baghdad, Iraq

**Keywords:** Traumatic events, PTSD, secondary school students

## Abstract

**Background:**

People in Iraq have been more or less continually exposed to war for more than three decades. Studies with Iraqi participants report high prevalence rates of posttraumatic stress disorder (PTSD) and related problems.

**Methods:**

The aim of this study is to measure the prevalence of traumatic events and to screen the prevalence of PTSD symptoms among Iraqi secondary school students. Four self-report scales were administered to 403 secondary school students, aged 16–19 (61% male and 31% female). These scales were Baghdad Trauma History Screen, the Scale of Posttraumatic Stress Symptoms (SPTSS), Social Support Scale, and Scale of Religious Coping.

**Results:**

The results showed that 84% of participants experienced at least one traumatic event. Of these, 61% fully met the criteria for PTSD; 65% of the females and 58% of the males. PTSD symptoms were correlated with 20 positive religious coping but not with social support.

**Conclusions:**

It’s clear that traumatic events were speared widely among the participants and the result showed that the vast majority of participants were exposed to different types of traumatic events. In addition, many of the participants have met full PTSD criteria and others had partial PTSD.

People in Iraq have been exposed to almost continual war for more than three decades: the Iraq–Iran war from 1980 to 1988; the chemical attacks in north Iraq from 1986 to 1989; the Gulf war in 1991; the civil strife of the early 1990s; and finally the war between coalition forces and Iraq, incorporating the civil war, from 2003 to 2011. Sectarian violence is on-going, still causing hundreds of—mainly civilian—fatalities a month. According to the Iraq Family Health Survey Study Group (IFHSSG, 2008), the worst year for casualties was 2006, with 223,000 fatalities, the majority of whom were male. A fifth of all casualties among Iraqi civilians were reportedly caused by approximately 1,000 suicide bombings between 2003 and 2010 (Hicks, Dardagan, Bagnall, Spagat, & Sloboda, 2011).

Given the high frequency of violent events in Iraq, high rates of posttraumatic stress disorder (PTSD) and other psychological disorders have been reported. Several studies have been conducted with Iraqi participants, both inside and outside Iraq, to examine traumatic events and their aftermath, as well as the prevalence of traumatic stress symptoms (Alezerjawi, 2005; Ashraf, 2004; Ghalib, 2004). Abdel-Hamid, Salim, AlQaisi, and Ahmad (2004) explored the prevalence of PTSD in Baghdad after the war in 2003 with a sample of 402 people (202 men and 200 women), aged 18–70. They found that 35.3% of participants reported symptoms of PTSD. Similarly, Al-Kubaisy and Alasdi (2004) examined the prevalence of PTSD symptoms. Participants consisted of 300 female Iraqi college students with an average age of 20.6 years. Overall 187 (62%) participants were exposed to at least one traumatic event; of these, 82% (155 of 187) reported that they experienced PTSD symptoms; 39% met the criteria for full PTSD, whereas 12% met the criteria for partial PTSD. A later study conducted on 284 Baghdad students (241 females, 43 males, aged 17–54) found that 196 (69%) experienced at least one traumatic incident. The most frequent traumatic events were sudden death of a family member (55%), bomb explosion (50%), seeing someone being killed or injured (37%), and killing of a family member (33%) (Al-Kubaisy, Hassan, & Al-Kubaisy, 2009).

A study of the prevalence of mental disorders in children by Razokhi, Taha, Taib, Sadik, and Gasseer (2006) compared three Iraqi cities. The results in Baghdad city showed that 47% of 600 primary school pupils in Baghdad (mean age 10.3 years) reported exposure to at least one traumatic event, whereas14% suffered from PTSD. In Mosul, a higher percentage of traumatic events were reported by adolescents aged 13–18. Thirty percent of 1,090 participants had PTSD symptoms. In addition, the older adolescents reported a higher rate of PTSD and only 8% of them had received treatment. In Dohuk, similar results were found when mental disorders were assessed in 240 children (120 working street children and 120 school children). The results showed that 36% of working street children and 16% of school children had mental disorders. The higher rate in working street children is unsurprising given their greater vulnerability or exposure to traumatic events (Razokhi et al., 2006).

Despite the traumatic events and the prevalence of PTSD in Iraq's population, mental health services in Iraq have been dramatically reduced since the 1980s. The number of workers in the mental health sector was one psychiatrist per 300,000 before 2003; by 2010, the number had decreased to one psychiatrist per 1 million (Sadik, Bradley, Al-Hasoon, & Jenkins, 2010). Many specialist doctors in Iraq emigrated because of the security risk between 2004 and 2007 (Burnham, Lafta, & Doocy, 2009). As a result, health services in Iraq are the poorest in the region (Tarantino, Morton, Kosaraju, Jawad, & Casscells, 2009), with the possible exception of occupied Palestine. In addition, because of the widespread violence, many families have lost one or more members. These families tend to seek support from sources other than professional therapy, because in Iraq it is not only difficult to obtain but also prohibitively expensive (Jaber, 2012).

Media coverage in Iraq has focused on the events in Iraq rather than on the victims, their feelings, and their ways of coping with trauma. In the light of this situation, traumatised people in Iraq do not have adequate access to mental health services that could help reduce these high levels of PTSD. Pargament (1997) has argued that religious coping may be more important for people with limited economic and social resources, offering them alternative solutions to life problems.

This study has two key aims; first, it elucidates the prevalence of traumatised people aged 16–19 as there is a lack of studies conducted on Iraqi participants of this age. Second, it provides an exploration of particular coping strategies through investigating the role of social support and religious coping.

## Study aims

The aims of this study are to investigate the following:

1. The type and number of traumatic events.
2. Prevalence of traumatic events according to gender.
3. The prevalence rate of PTSD symptoms.
4. The relationship between PTSD symptoms and religious coping and social support.

## Methods

### Participants

Students aged 16–19 from four schools in Baghdad, two male-only and two female-only, were invited to take part in the study. Out of 450 students contacted, 403 consented to participate: 248 (61.5%) males and 155 (38.5%) females.

### Measurements

Four scales were administered: the Baghdad Trauma History Screen, the Scale of Posttraumatic Stress Symptoms (SPTSS), the Social Support Scale, and the Scale of Religious Coping. These scales were administered at the participants’ schools between December and January 2011. This study was approved by the ethics committee of the Institute of Work, Health and Organisations, University of Nottingham.

### Baghdad Trauma History Screen

Developed by Jaber (2012), this scale lists 21 traumatic events. The participant should determine first if he or she was exposed personally and/or whether a family member or friend was involved, followed by the age at which the event occurred and its frequency of occurrence. Finally, the respondent indicates whether he or she felt fear, horror, or helplessness as a result of the experience.

### The Scale of Posttraumatic Stress Symptoms

This scale has been validated in the Iraqi population (Jaber, 2012). The SPTSS uses a five-point scale (0= not at all, 1= once or twice, 2= almost every day, 3= once a day, and 4= more than once a day). The scale has 17 items and is matched to the diagnostic criteria for PTSD in DSM-IV. It is used to screen and estimate symptom occurrence during the previous month. For the purposes of classifying probable PTSD and probable partial PTSD, participants have to indicate 1) one or more of the five re-experiencing items, 2) three or more of the seven avoidance items, and 3) two or more of the five arousal items. Overall scale scores were used for classification as probable PTSD.

There were several reasons to use this scale; first, this scale was validated with young people aged 18–22, which is similar to the age of the current participants. Second, the SPTSS has been used by psychiatrists to screen PTSD symptoms in Iraqi refugees in Jordan (though the results are not yet published). Third, a discussion with Iraqi psychiatrists indicated that using this scale could be helpful to screen PTSD symptoms in the Iraqi population, particularly with people who have been exposed to multiple and continuing traumatic events.

Internal consistency was assessed using Cronbach's alpha, with scores of 0.90, 0.84, 0.82, and 0.67 for the total scale, and the re-experiencing, avoidance, and hyperarousal subscales, respectively. Test–retest reliability scores were 0.83, 0.80, 0.78, and 0.77 for the total scale, and the re-experiencing, avoidance, and hyperarousal subscales, respectively.

### The Social Support Scale

Developed by Jaber (2012), this four-point scale was validated in the Iraqi population. The scale contains 14 items and measures three sources of social support: family, friends, and governmental and non-governmental organisations (GNGO). Regarding reliability, Cronbach's alpha was 0.95, 0.97, and 0.95 for family, friends, and GNGO items, respectively.

### Religious coping (The brief RCOPE)

With a four-point scale, Brief RCOPE is a 14-item questionnaire (Pargament, Feuille, & Burdzy, 2011) assessing two styles of religious coping: positive and negative. There are seven positive items (e.g., looked for a stronger connection with God) and seven negative items (e.g., I wondered what I did for God to punish me). The scale was translated from English to Arabic, and back-translated by a different interpreter. All items were reviewed by five psychologists in Baghdad universities. Cronbach's alpha was conducted to examine internal consistency, and the scores were: 0.86 for positive and 0.82 for negative subscales.

## Results

### Number of traumatic events

Based on the data collected regarding trauma history, participants reported a range of traumatic events with a range of frequencies. These were either experienced by self or experienced by other. Table 1 shows that 84.4% reported experiencing at least one traumatic event.

**Table 1:** Number of participants according to frequency of traumatic events and type of exposure (*N*=403)

	Number of traumatic events				
		
	1–5 *N* (%)	6–10 *N* (%)	11–15 *N* (%)	16 or more *N* (%)	Total
Other and/or self exposed	90 (22.3)	100 (24.8)	33 (8.2)	6 (1.5)	229 (56.8%)
Self exposed	78 (19.3)	5 (1.2)	1 (0.2)	0 (0)	84 (20.8%)
Other exposed	26 (6.4)	1 (0.2)	0 (0)	0 (0)	27 (6.7%)
Total	194 (48.1)	106 (26.3)	34 (8.4)	6 (1.5)	340 (84.4%)

### Exposure to traumatic events

The results showed that 340 of participants were exposed to at least one traumatic event. The types of exposure were categorised into three groups: self-exposure (SE), other exposure (OE), and self and other exposure (SOE). Table 2 shows that most participants reported SOE, with only around a quarter reporting traumatic events that were SE. Fewer than 8% reported only OE. With regard to gender, the results showed a significant difference (*χ*^2^=12.38, *p*<0.01), with males reporting more traumatic events than females.

**Table 2:** Prevalence of traumatic events according to gender

Type of exposure	Self and other	Self only	Others only	Total	*χ*^2^
Gender					
Male	128 (62.4%)	64 (31.2%)	13 (6.3%)	205 (100%)	12.38*
Female	101 (74.8%)	20 (14.8%)	14 (10.4%)	135 (100%)	
Total	229 (67.4%)	84 (24.7%)	27 (7.9%)	340 (100%)	

* *p*<0.05.

### The prevalence rate of PTSD symptoms

The three PTSD criteria (re-experiencing, avoidance, and hyperarousal) were used to classify the reported symptoms. The categories are full PTSD (meets all three criteria) and partial PTSD (meets one or two criteria). Table 3 shows the prevalence of PTSD symptoms among the participants. The results show that around 92% of those exposed to traumatic events had at least some PTSD symptoms, with 61.5% meeting the criteria for full PTSD. In terms of gender, the proportion of females who fully met PTSD criteria was significantly higher than males (*χ*^2^=9.408, *p*<0.001).

**Table 3:** Prevalence of PTSD according to gender and categories of PTSD

	PTSD			
		
Sex	Fully, *n* (%)	Partially, *n* (%)	None, *n* (%)	Total
Males	120 (58.5)	62 (30.2)	23 (11.2)	205 (60.3%)
Females	89 (65.9)	43 (31.8)	3 (2.2)	135 (39.7%)
Total	209 (61.5)	105 (30.9)	26 (7.6)	340 (100%)

### The relationship between PTSD symptoms and religious coping and social support

The relationship between PTSD and religious coping and social support was examined. The Pearson correlations are presented in Table 4. The results showed that there were no significant relationships between PTSD scores and most variables, except the positive subscale of religious coping (*r =*0.118, *p*<0.05).

**Table 4:** Relationships between number of PTSD symptoms and religious coping and social support

Variables	Correlation with PTSD score
Religious coping	0.053
Positive sub scale of religious coping	0.118*
Negative sub scale of religious coping	0.018
Social support received from family	−0.038
Social support received from friends	0.010
Social support received from GNGO	0.072

* *p*<0.05.

## Discussion

The results showed a high rate of prevalence of experiencing traumatic events among Iraqi secondary school students. Participants reported the highest percentage of exposure to traumatic events in the “experienced by self and others” category. Males were more likely to report SE to traumatic events than females. Participants reported varying levels of PTSD symptoms, with the majority meeting the full set of criteria for PTSD. This finding is consistent with several studies conducted in Iraq on adults and adolescents (Abdel-Hamid et al., 2004; Al-Kubaisy & Alasdi, 2004; Al-Kubaisy et al., 2009; Alezerjawi, 2005; Ashraf, 2004; Dyregrov, Gjestad, & Raundalen, 2002). Females suffered more PTSD symptoms than males. These results are supported by Ghalib (2004) and Jaber (2012).

Regarding religious coping, only the positive religious subscale was significantly negatively correlated with PTSD symptoms. According to Islamic culture, religion plays an important role and is used widely for dealing with life's difficulties (Aflakseir & Coleman, 2011). Muslims often perceive God to be helping them after they have been exposed to traumatic situations. Furthermore, they seek to be closer to God, which in turn prompts them to feel more comfortable, and this may contribute to reduced PTSD symptoms. This result is consistent with previous studies which have demonstrated that positive religious coping is negatively associated with PTSD (Fallot & Heckman, 2005; Meisenhelder & Marcum, 2004; Pargament, Smith, Koenig, & Perez, 1998).

Pargament et al. (1998) suggest positive religious coping methods are an expression of a sense of spirituality, a secure relationship with God, a belief that there is meaning to be found in life, and a sense of spiritual connectedness with others. In contrast, a negative religious coping pattern is an expression of a less secure relationship with God, a tenuous and ominous view of the world, and a religious struggle in the search for significance, which may help explain why those with high levels of positive religious coping have lower levels of PTSD.

There was no relationship between PTSD symptoms and any of the sources of social support (family, friends, and GNGOs), suggesting that these may not be effective in helping traumatised people to recover, perhaps because of the severity and prevalence of the trauma in Iraq; people may be suffering so much they are unable to help others. Other studies have found social support is not correlated with PTSD (Andrews, Brewin, & Rose, 2003; Gold et al., 2000; Zoellner, Foa, & Brigidi, 1999).

There are limitations to the study. The results are based on data collected only from secondary school students in Baghdad. This limits the generalisation of these results to the general population. These students have only known war. Another issue is that the findings are based on self-report, rather than clinical assessments. This was partly because of the difficulties of obtaining data in Baghdad, which remains a dangerous city.

In conclusion, the findings suggest that most people in this population of Iraqi school children have experienced traumatic events, and that most, particularly young women, are traumatised by these experiences. Unfortunately the experiences are on-going, and so the levels of PTSD are likely to rise. Levels of social support are not associated with PTSD, suggesting that people have limited ability to offer or receive such support in Iraq, an area needing more research. The only relationship was between PTSD and positive religious coping, suggesting those who have a clear and positive attitude regarding religion are more able to deal with their trauma symptoms. Future research should also examine the role of religion as a coping mechanism.
